# Investigating the role of mood induction on emotional facial recognition in social anxiety

**DOI:** 10.1371/journal.pone.0332748

**Published:** 2025-09-25

**Authors:** Corina Lacombe, Kassia Dubé, Charles Collin

**Affiliations:** School of Psychology, University of Ottawa, Ottawa, Ontario, Canada; Gabriele d'Annunzio University of Chieti and Pescara: Universita degli Studi Gabriele d'Annunzio Chieti Pescara, ITALY

## Abstract

Individuals with high trait social anxiety (SA) experience multiple challenges when interacting with others. Social skills abilities like accurate emotional facial expression recognition are particularly impaired in this population. Ambiguous and angry facial expressions are most often miscategorized and met with uncertainty. Part of this confusion may be attributable to increased state anxiety when approaching social situations. However, little is known about the influencing role of state anxiety on emotional facial expression recognition among those with social anxiety. The present study aimed to evaluate the impact of state anxiety on emotional facial recognition. Sixty-eight undergraduate students with high trait social anxiety participated in a pre-post emotional facial recognition task. Participants were presented with happy, neutral, and angry facial expressions in random order and asked to categorize the expressed emotion among six basic emotion categories. In between emotional facial recognition tasks, participants engaged in a mood induction procedure (i.e., mock discussion with a confederate) aimed to increase state anxiety. The results suggest that individuals with high-trait SA were significantly worse at recognizing happy facial expressions post-affect induction. Furthermore, individuals with high-trait SA showed significant difficulty in accurately recognizing neutral facial expressions across pre- and post-conditions. An error rate analysis revealed that neutral and happy facial expressions were most often miscategorized as either surprise, angry, sad, or disgust. This study highlights that positively-valenced expressions are met with increased uncertainty particularly when experiencing elevations in state anxiety.

## Introduction

Emotional facial recognition is a non-verbal behaviour that facilitates social interaction [1–3]. Feedback from emotional facial expressions can help an observer gauge whether to approach or avoid a social situation [3–4]. For instance, a fearful facial expression, which is prototypically conveyed through widened eyes, is an adaptive behavioural response to signal a threat. Similarly, a facial expression of disgust, normatively characterized by a wrinkled nose and raised upper lip, can signal the presence of a poisonous or toxic stimulus. Alternatively, observing a smile can elicit prosocial behaviours and enhance social connection [5]. Accurately recognizing facial expressions of emotion is, therefore, an evolutionarily adaptive skill necessary for avoiding potential danger [6–7]. In social contexts, these dangers include aversive experiences like rejection [8], which can result in isolation and feelings of loneliness [9]. Thus, accurately recognizing emotional facial expressions is a key component of approaching and fostering interpersonal relationships [10–11]. However, individuals who have deficits in their ability to accurately recognize emotional facial expressions are at risk of missing out on these social benefits and even worse, may misperceive them as more threatening, thus perpetuating avoidance. Hudd and Moscovitch (2020) [12] point out that individuals with high sensitivity to social rejection, like those with high trait social anxiety (SA), have a decreased ability to perceive socially rewarding cues from their environments. These cues, like happy facial expressions, may be perceived by those with social anxiety disorder (SAD) as untrustworthy, contemptuous, or as mocking [13–14]. A recent meta-analysis by our group confirms that individuals with a primary diagnosis of SAD were significantly worse at recognizing happy and neutral facial expressions compared to healthy controls [15].

There are multiple reasons why facial expressions might be perceived more negatively among those with SAD. Rapee and Heimberg (1997) note that individuals with SAD allocate a great deal of attentional resources to monitoring for potentially threatening external indicators. In a population that fears negative evaluation from others, indicators like frowns, signs of boredom, or other ambiguous facial expressions can elicit impairing and distressing emotional, somatic, cognitive, and behavioural symptoms of anxiety [2]. These aversive symptoms subsequently lead to the use of safety behaviours and avoidance, like avoiding eye contact [16]. The consequences of engaging in gaze avoidance of crucial parts of the face when interacting with others are pronounced. It has not only been shown to decrease emotional facial recognition accuracy but also to limit the ability to integrate corrective feedback and thus modify maladaptive beliefs [17–19]. This may lead to a negative cycle whereby avoidance of facial cues leads to a poorer ability to interpret them, which in turn leads to greater difficulties in social interaction. This cycle maintains the disorder over time as it reinforces erroneous beliefs, thoughts (e.g., “I will be rejected”) and behaviours like hypervigilance and excessive monitoring for threat cues [2,20,21]. As a result, individuals with social anxiety often approach social situations in an already anxious state, which may worsen their emotional facial recognition abilities.

The Emotions as Social Information (EASI) model of emotion recognition posits that inferential processing of emotional facial expressions is mediated by the affective state of the observer. Authors van Kleef and Côté (2022) theorize that an observer can infer other’s emotional expressions based on their *own affective reaction* to the expressor. Therefore, an individual that enters a social interaction in an already anxious state may be biased in interpreting the expressors emotion. A study by Dyer et al. (2021) [22] investigated the impact of state anxiety on emotional facial recognition. By manipulating carbon dioxide (CO_2_) intake concentrations, the authors were able to experimentally increase state anxiety before engaging in a 6-alternative forced choice emotional facial recognition task. Their results indicated that high state anxiety significantly worsened emotion recognition abilities. Mood induction paradigms have also been used to temporarily alter affective state. Schmid and Mast (2010) [23] found that negative mood induction through emotionally evocative video clips enhanced emotional facial recognition for angry and happy facial expressions among healthy participants. Manierka et al.’s (2021) [24] results also suggested that positive mood induction among a healthy sample decreased accuracy in recognizing happy facial expressions.

As it relates to social anxiety, studies have successfully induced an anxious state in individuals with high trait SA by asking participants to partake in a mock evaluative conversation task with a confederate [25–26]. In both Kelly-Turner and Radomsky (2020) and Ferguson et al. (2023), the authors asked participants to engage in a time-limited conversation where the confederate asked participants questions about themselves (e.g., “tell me about yourself”) before engaging in a task. State anxiety was monitored by asking participants to rate their current level of distress on the Subjective Units of Distress Scale (SUDS) at various points throughout their experiments.

In the current study, we aimed to use a similar mood induction paradigm to evaluate the effect of state anxiety on emotional facial recognition abilities in a university sample with high trait SA. We conducted a pre-post Facial Emotion Recognition (FER) task; the FER task was separated by a time-limited discussion with a confederate. The conversation task was adapted from Stopa and Clark (1993). Furthermore, we informed participants that their conversation would be video recorded, and that the quality of their response would be self-evaluated and evaluated by the confederate. This was done to increase the effectiveness of our mood induction [27]. To obtain a complete understanding of emotional facial recognition abilities, we assessed three FER outcome measures both pre- and post-conversation: accuracy, intensity, and saliency.

Accuracy simply measures whether a participant sees the target emotion (i.e., the emotion facial expression the model is trying to portray) in an image as the most intense one, regardless of what other emotions might be mixed in with it. This measure is important because it determines the category into which a participant would place an emotional expression if forced to choose just one, and allows us to evaluate if they do so correctly. However, it is a limited measure because it ignores the fact that emotional expressions can vary in intensity and can portray mixtures of emotions. For this reason, we also measured the intensity with which each emotional expression was evaluated, as well as saliency, a measure of the degree to which the target emotion was perceived to appear purely on its own, as opposed to mixed with other emotions. Based on previous research, we developed the following hypotheses:

H1: Participants would report a greater subjective anticipatory and post-event anxiety on the Subjective Units of Distress Scale compared to baseline (*manipulation check*).

H2: Participants would do more poorly in categorizing facial expressions post-conversation than pre-conversation. This would be reflected in lower accuracy.

H3: Participants would interpret emotional facial expressions less clearly post-conversation than pre-conversation. This would be reflected in lower intensity and saliency values.

H4: Participants would rate their performance during the conversation task more negatively than the confederates using the Social Performance Rating Scale.

## Method

This study was approved by the University of Ottawa’s Office of Research Ethics and Integrity (H-02-23-8878) on April 3, 2023.

### Study design

The primary aim of this study was to evaluate whether emotional facial recognition performance would worsen following a mood-induction procedure (MIP). A 2x3 repeated measures ANCOVA with emotional facial expression as the dependent variable (3 levels: angry, happy, neutral), state anxiety as the independent variable (2 levels: pre and post-MIP), and with trait SA as a covariate was conducted for each of our three outcome measures (i.e., accuracy, saliency, intensity). In the emotion recognition task, participants were presented with 60 trials in the pre-MIP and 60-trials in the post-MIP (totalling 120 trials). In each of the trials, participants were presented with either an angry, happy, or neutral facial expressions and asked to categorize them accordingly. A detailed diagram of the study procedure can be seen in Fig 2.

**Fig 1:**
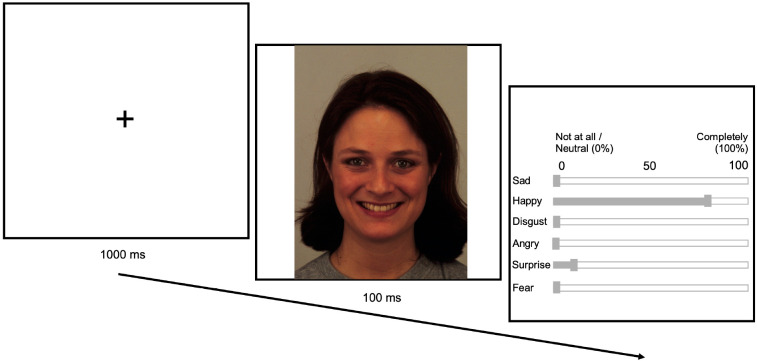
Sample facial emotion expression rating trial. The facial expression image displayed in the sample trial is taken from the Karolinska Directed Emotional Faces database.

### Participants

#### Sample size considerations.

Dyer and colleagues (2021) were able to observe a significant effect of elevated state anxiety on performance in emotional facial recognition tasks in a sample with high trait SA with effect sizes ranging from d = 0.29 to d = 0.49. For the current study, we conducted a power analysis (d* *= 0.50, *α *= 0.05, power = 0.95) using G*Power [28] in line with the upper range of Dyer et al.’s (2021) effect sizes. For our most stringent analysis, a sample size of **n* *= 54 was suggested. To account for possible technology malfunctions, participant attrition, or discovery of the confederate, we supplemented our sample size by 30%, recruiting a sample of 68 participants.

#### Recruitment.

The sample consisted of undergraduate students who were recruited through the University of Ottawa’s Integrated System for Participation in Research. Participants received one-course credit as compensation. Recruitment occurred between September 15, 2023 and April 2, 2024. Participants were invited to engage in an in-person computer-based emotional facial recognition task taking place at the University of Ottawa. Inclusion criteria included normal or corrected-to-normal vision and no history of a severe psychiatric disorder (e.g., bipolar disorder, schizophrenia-spectrum disorder). All participants met the listed inclusion criteria. Additionally, participants were provided with a baseline questionnaire package, which included a sociodemographic questionnaire, the Liebowitz Social Anxiety Scale – Self-Report (LSAS-SR) to assess for trait SA, and the Depression and Anxiety Stress Scale (DASS-21). Given that this study focused on evaluating the impact of state anxiety within a sample with high trait SA, only participants who had an LSAS-SR score of 30 or above were included in the final analyses. A clinical sample was not used in this study, as research has shown that individuals with clinically elevated anxiety generally have already elevated levels of state and trait anxiety. As such, using a non-clinical sample would allow us to assess the unique influence of state anxiety in a sample with varying degrees of elevated trait SA [29,30]. Participants who had an LSAS-SR score of 29 or below (*n* = 8), had more than 5% of their data missing (*n* = 1), or disclosed that they were aware they were interacting with a confederate (*n* = 7) were excluded.

### Measures

#### Sociodemographic questionnaire.

Participants were asked to provide information relating to their demographic characteristics (i.e., age, gender identification, ethnicity identification, employment status, dominant language, history of a mental health disorder). A summary of the sample characteristics can be found in the Results section.

#### Liebowitz social anxiety scale – self-report.

The self-report Liebowitz Social Anxiety Scale is a 24-item self-rated questionnaire that assesses key components of social anxiety (fear and avoidance) experienced by the participant over the past week. Items on the fear dimension are individually scored on a four-point rating scale ranging from 0 (none) to 3 (severe) [31]. Similarly, avoidance is rated on a four-point rating scale ranging from 0 (never, 0%) to 3 (usually, 68–100%). Scores that range from 0–29 indicate no/low social anxiety, 30–59 indicate nongeneralized social anxiety, and scores of 60 + indicate generalized social anxiety [32]. The current sample was screened using the LSAS-SR to assess for symptoms of social anxiety. We sought to only include the data of participants who had a total score of 30 and above. The test reported excellent internal consistency for the fear and avoidance subscales [33]. The internal consistencies for the fear (ω = 0.886, 95% CI = [0.827, 0.992]) and avoidance (ω = 0.812, 95% CI = [0.712, 0.866]) subscales in the current sample were likewise excellent.

#### Depression anxiety and stress scale–21 (DASS-21).

The DASS-21 is a 21-item self-report that assesses symptoms of depression, anxiety, and stress experienced over the past week. Items on each of the subscales are individually scored on a four-point rating scale ranging from 0 (did not apply to me at all) to 3 (applied to me very much, or most of the time). The internal consistencies for all subscales were excellent [34]. Within our sample, the internal consistencies for depression (ω = 0.804, 95% CI = [0.716, 0.870]), anxiety (ω = 0.828, 95% CI = [0.701, 0.899]), and stress (ω = 0.806, 95% CI = [0.697, 0.874]) were very good. This scale was included as part of a larger project and its outcomes were not analysed for the purposes of the present study.

#### Subjective units of distress scale (SUDS).

The Subjective Units of Distress Scale is a measure used to assess participants’ subjective level of state distress [35]. Similar to other studies, we modified the measure to evaluate participants’ state level of anxiety, rather than distress [25]. Participants rated their anxiety on a sliding scale ranging from 0 (no anxiety) to 100 (extremely anxious).

#### Social performance rating scale (SPRS).

We used the five-item Social Performance Rating Scale (SPRS) to assess the consistency of the confederates’ behaviour across participants. Two research assistants blind to the study aims and hypotheses were asked to rate the confederate’s gaze, vocal quality, length of statements, overt signs of discomfort, and conversation flow during the video-recorded conversation task. Each component was rated on a five-point Likert scale ranging from 1 (very poor) to 5 (very good) [36]. The SPRS has been used in conversation tasks with similar mood induction paradigms [26,37]. Similar to other studies, we assessed inter-rater reliability by averaging the SPRS scores and calculating the intraclass correlation coefficient (ICC) to determine inter-rater reliability [38]. Our ICC suggests good reliability (ICC = 0.81, 95% CI = [0.67, 0.89]) [39].

#### Behaviour rating scale.

Given that individuals with social anxiety tend to misperceive their own performance in social interactions, we asked participants to rate how they perceived their performance during the discussion with the confederate [40]. Participants were asked to rate the 23-item checklist indicating how characteristic their performance was on a series of 16-positive (e.g., friendly, relaxed, warm, confident, assertive) and 7-negative (e.g., nervous, blushing, hands shaking) behaviours (see supplementary materials for appended checklist). The 9-point rating ranged from 0 (not characteristic at all) to 8 (extremely characteristic). Participants were also asked to repeat this checklist, rating the confederate’s behaviour.

#### Credibility questionnaire.

As recommended in Kelly-Turner and Radomsky (2020), we compiled a ‘believability’ score to evaluate how much participants believed that they were being evaluated, and whether they knew the other participant to be a confederate. Participants were asked to rate this on a sliding scale ranging from 0 (I did not believe I was being evaluated) to 100 (I believed that I was being evaluated). Similarly, participants were asked to indicate ‘yes’ or ‘no’ to whether they believed to be interacting with a confederate during the discussion task.

### Emotional facial expression rating tasks

Participants engaged in two facial emotion recognition (FER) tasks (i.e., pre- and post-mood induction procedure), each consisting of 60 trials (20 trials for each of the three emotional facial expressions presented). Trials contained an image of either an angry, happy, or neutral facial expression displayed for 100ms, followed by a 1000ms intertrial interval of a fixation cross, and an emotion categorization question. Specifically, participants were provided with six slider scales representing the Ekman emotional facial categories (anger, surprise, fear, sadness, happy, disgust), and asked to rate on a scale of 0 (neutral) to 100 (emotion) what emotion(s) they perceived [41] (see Fig 1). A sample trial was shown to participants prior to commencing the experiment to familiarize themselves with the task. In the sample trial, participants were informed that they would be presented with and instructed on how to use the six slider scales. Participants were not informed which emotional facial expressions would be presented as this may have biased the emotion categorization task.

The stimuli consisted of 120 frontal images of facial expressions of emotion from the Karolinska Directed Emotional Faces (KDEF) database [42]. Images with the highest accuracy scores from image set A of the KDEF database was used because it has been previously validated. All models were young male and female white adults between the ages of 20–30 with no identifying features (e.g., facial hair, makeup, markings, glasses, or tattoos). The same model was presented three times, once depicting each facial expression (happy, neutral, angry) to minimize any effects that may be due to the individual differences in the model’s appearance. The three emotional categories were chosen because previous research suggests that individuals with social anxiety tend to miscategorise threatening, happy and neutral expressions [15]. Different stimuli were used and counterbalanced in the pre and post FER tasks to reduce order effects.

#### Conversation task.

The recruitment study advertisement posted in the University of Ottawa’s Integrated System for Participation in Research stated that participants would be asked to participate in a brief discussion with another participant (i.e., trained white male confederate). In the process of discussing informed consent, experimenters once more informed participants that they would be engaging in a 5-minute discussion with another participant on a particular hypothetical topic, and that their conversation would be video recorded. The purpose and content of the discussion was not disclosed at any point prior to commencing the experiment. To ensure consistency, a single confederate was used across all participants. At the time of the discussion, and the participant and confederate were reminded that their discussion would be video recorded and were informed that they would be evaluating each other’s responses to the discussion topic. Discussion topics were adapted from Stopa and Clark (1993) where the confederate was asked to read out loud a hypothetical situation and asked participants “what would you do in this situation?’. These situations included (1) “*You have just started a new job and you have to give a short talk about yourself and about why you wanted the job. Your audience is a group of six people who already work there*”; (2) “*You have gone to the pub with an acquaintance. Your acquaintance sees two old friends whom you do not know and were not expecting to meet. You all sit down together*”; and (3) “*You have bought something at a shop and found a fault. You have to ring the manager to complain and to ask for a replacement*.” Similar to Kelly-Turner and Radomsky (2020), the confederate was trained to initiate the conversation, and to appear warm and interested. To reduce order effects, discussion topics were counterbalanced across participants.

### Procedure

This study was completed in-person and approved by the University of Ottawa’s Research Ethics Board (H-02-23-8878). Prior to obtaining written consent, participants were informed that they would be conducting two emotion recognition tasks and participating in a peer discussion with another participant, which would be video recorded. The experimenter provided the participant with a sample FER trial and addressed any task-related questions. Participants then completed a Sociodemographic Questionnaire, a baseline measure of state anxiety using the SUDS and baseline questionnaire package assessing trait SA using the LSAS-SR and symptoms of depression, anxiety, and stress using the DASS-21. Prior to beginning the first Facial Emotion Recognition (FER) task, participants were asked to complete a second SUDS. Once the participant completed the first FER task, the researcher informed them that they would begin the peer discussion shortly. At this time, participants completed a third SUDS to evaluate anticipatory state anxiety. Once the confederate and participant were joined in the same room, the researcher presented them with the discussion topic and reminded both that they would be video recorded and would need to evaluate the quality of theirs and the other’s responses to the question. Once the 5-minute discussion was completed, the participant returned to their computer and were asked to complete a fourth SUDS to assess post-event anxiety and the behaviour checklist, followed by the final FER task.

Following the task completion, the researcher thoroughly debriefed the participant, and consent was re-obtained given that mild deception had been used. Finally, participants were asked to complete a final SUDS to assess anxiety levels after the debrief, and a 2-item credibility questionnaire to assess the believability of the deception. Specifically, participants were asked how much they believed that they were being evaluated during the discussion and whether they knew the other participant was indeed a confederate.

### Statistical analyses

The data of seven participants was not included in our analyses because they reported knowing that they were conversing with a confederate. Similarly, list-wise deletion was used to remove the data of an eighth participant who had more than 5% of their data missing. An additional eight participants were removed from the analyses, as their trait SA scores measured through the LSAS-SR scores fell below the clinical cut-off score. As a result, the data from 52 participants was used in all of the listed analyses. The distribution of scores in histograms, QQ plots, and residual plots were visually inspected and revealed no apparent deviations from normality. Skewness and kurtosis were further evaluated to assess for deviations from normality. Ceiling effects were observed for the saliency and accuracy of happy facial expressions, which was reflected in a negatively skewed distribution. No spurious univariate or multivariate outliers were identified in the sample. The demographic sample characteristics can be found in Table 1.

**Table 1:** Participant demographic characteristics.

Variable	%	*M*	*SD*	Range
**LSAS-SR score**		58.92	19.7	31–110
**DASS-21 score**				
Depression		24.46	7.60	14–44
Anxiety		24.00	8.79	14–54
Stress		27.69	7.71	14–44
**Age**		19.42	3.04	16–34
**Gender**				
Cisgender woman	69.23			
Cisgender man	15.29			
Female	11.54			
Male	3.85			
**Ethnicity**				
Arab	5.77			
Arab/African	1.92			
Asian (East, South, Southeast, Central, Other)	19.23			
Black – African	11.54			
Black – Caribbean	5.77			
First Nations	1.92			
Latin American	1.92			
Middle Eastern	3.85			
Mixed Heritage	1.92			
White – European	25.0			
White – North American	15.39			
Other (nothing applies to me)	5.77			
**Occupation**				
Full-time student	98.08			
Part-time student	1.93			
**Received mental health diagnosis by a health professional**				
Yes	17.31			
No	80.77			
I prefer not to say	1.92			

**N* *= 52. *Note. LSAS-SR* = Liebowitz Social Anxiety Scale – Self Report; *DASS-21 =* Depression, Anxiety, Stress Scale.

The primary aim of this study was to evaluate whether state anxiety, measured through the SUDS, would influence emotional facial expression recognition performance while accounting for trait SA. To ensure that the manipulation was effective, data from the SUDS were analyzed using a repeated measures ANOVA. Data from the emotional facial recognition task were analyzed using repeated measures ANCOVAs with emotional facial expression as the dependent variable (3 levels: anger, happy, neutral), state anxiety as the independent variable (2 levels: pre and post-mood induction), and with trait SA as a covariate. A secondary aim of this study was to assess whether participants would rate their performance during the conversation task more negatively than the confederates using the Social Performance Rating Scale. Data were analyzed using a repeated measures ANOVA.

Greenhouse-Geisser corrections for violations of sphericity were used with the appropriate degrees of freedom. Effect sizes (i.e., partial eta-squared) are reported where appropriate. All analyses were conducted with SPSS Version 29 and JASP Version 0.18. Results of the ANOVAs are given in the sections below.

## Results

### Manipulation check

Using the SUDS, we evaluated participants subjective level of state anxiety at five points: baseline (*M* = 20.06, *SD* = 20.735), pre-emotion recognition task (*M* = 18.19, *SD* = 18.568), in anticipation of the discussion with a confederate (*M* = 22.06, *SD* = 21.805), post discussion (*M* = 20.29, *SD* = 20.920), and at debrief (*M* = 17.94, *SD* = 19.552). Across all timepoints, participants rated mild state anxiety. Results of a 1-way repeated measures ANOVA suggest that our manipulation did not significantly increase state anxiety at any of these time points (*F*(2.4, 122.403) = 1.336, **p* *= .267, η_p_^2^ = .026). These results suggest that our manipulation was ineffective at increasing state anxiety (see Fig 3).

**Fig 2:**
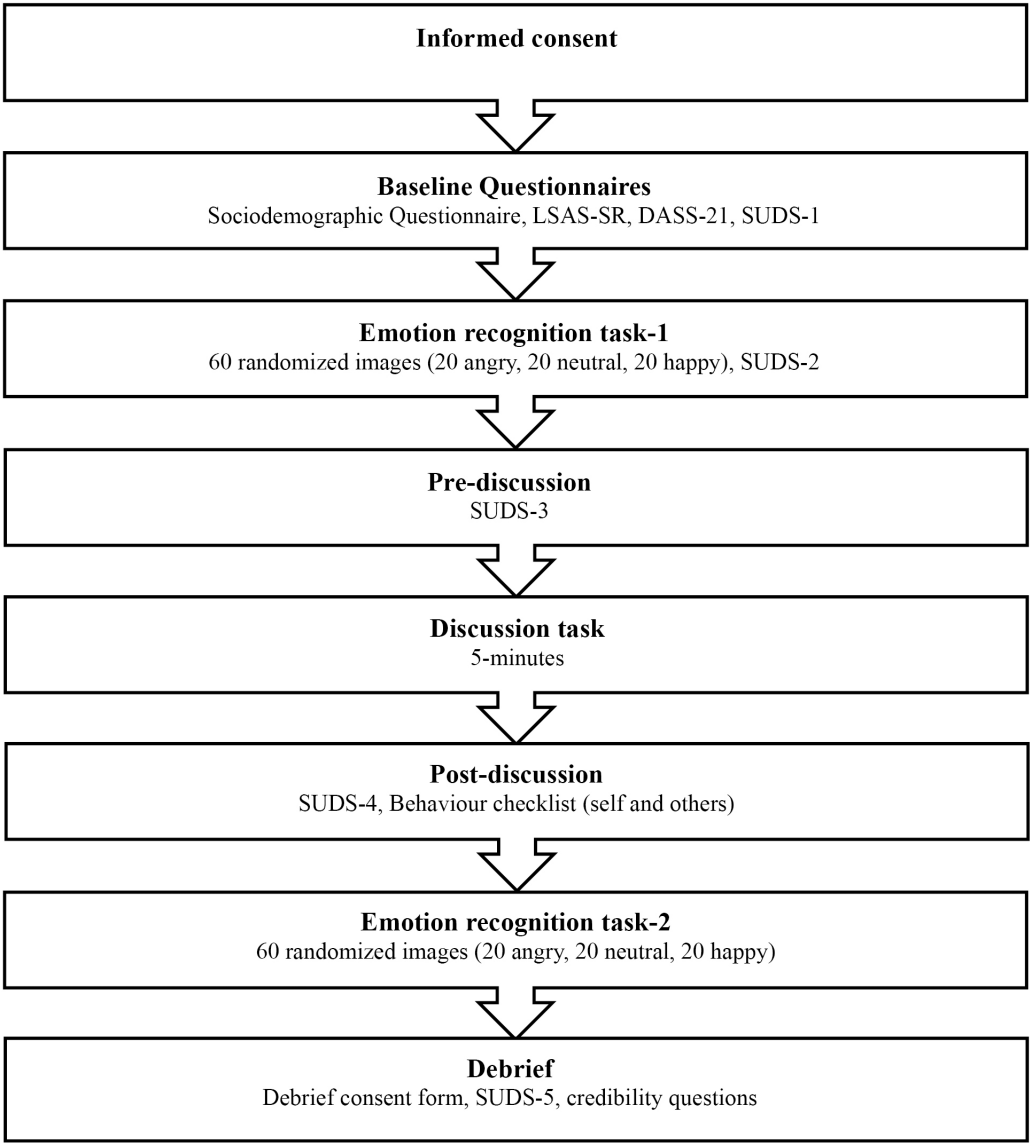
Procedural flow chart. LSAS-SR: Liebowitz Social Anxiety Scale – Self-Report; DASS-21: Depression, Anxiety, Stress Scale; SUDS: Subjective Units of Distress Scale.

**Fig 3:**
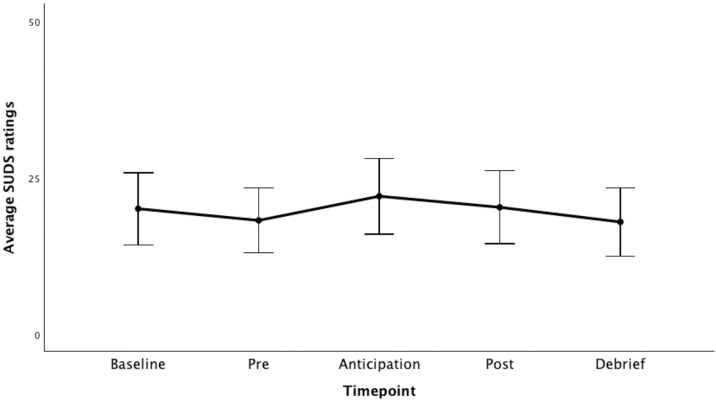
Subjective Units of Distress (SUDS) ratings across study timepoints. Mean ratings are encompassed by 95% confidence intervals.

### Emotional facial recognition

A repeated measures ANCOVA was conducted for each of the three-outcome measures (accuracy, intensity, saliency) and emotional facial expression (angry, happy, neutral). Intensity and saliency were not evaluated for the neutral emotion given that we theoretically could not assess how intense or salient the absence of an emotion is. The unadjusted and adjusted means listed in Table 2 indicate that the covariate trait SA had little to no influence on our outcome measures. Our main results were analyzed using a 2x3 ANCOVA for accuracy, and two 2x2 ANCOVAs for saliency and intensity. Main effects were observed for emotional facial expression across outcomes measures for accuracy (*F*(2, 50) = 20.465, *p* = < .001, η_p_^2^ = .290), intensity (*F*(2, 50) = 22.645, *p* = < .001, η_p_^2^ = .312), and saliency (*F*(2, 50) = 33.844, *p* = < .001, η_p_^2^ = .404). No interactions were observed between emotional facial expression and state anxiety (pre and post-mood induction). These results were followed up with a series of seven repeated measures one-factor ANCOVAs with 2 levels (pre- vs. post-conversation). An ANCOVA was conducted for each emotional facial expression (happy, neutral, angry) across all three outcome measures (accuracy, intensity, saliency), while using trait SA scores as the covariate. Our results suggest that participants were significantly worse at recognizing happy facial expressions post-mood induction (*F*(1, 50) = 4.367, **p* *= .042, η_p_^2^ = .080). No significant differences were observed between pre-post FER performance for the neutral or angry emotion categories (see Table 3). Similarly, no significant interactions were observed between the trait SA covariate and any of the outcome measures across all emotion categories (**p* *> .05).

**Table 2:** Unadjusted and covariate adjusted descriptive statistics for pre-post emotion recognition task.

Outcomes measures	Unadjusted		Adjusted	
	*M(SD)*_*pre*_	*M(SD)*_*post*_	*M(SD)*_*pre*_	*M(SD)*_*post*_
*Accuracy*				
Happy	0.911(0.073)	0.905(0.096)	0.911(0.072)	0.905(0.094)
Neutral	0.223(0.242)	0.264(0.247)	0.223(0.245)	0.264(0.245)
Angry	0.653(0.161)	0.668(0.174)	0.654(0.159)	0.668(0.173)
*Intensity*				
Happy	76.501(14.206)	71.951(15.738)	76.501(14.206)	71.951(15.706)
Angry	54.771(12.756)	54.318(14.779)	54.771(12.850)	54.318(14.920)
*Saliency*				
Happy	0.848(0.109)	0.831(0.135)	0.848(0.108)	0.831(0.137)
Angry	0.576(0.154)	0.600(0.161)	0.576(0.159)	0.600(0.159)

*N* = 52. *Note.* The adjusted means reported have been adjusted for the covariate, social anxiety, measured using the Liebowitz Social Anxiety Scale – Self-Report.

**Table 3:** Main effect estimates for pre-post emotion recognition task.

Outcome measures	*F*(1, 50)	*p*	*η*_*p*_^*2*^
*Accuracy*			
Happy	4.367	.042*	.080
Neutral	1.269	.265	.025
Angry	0.852	.360	.017
*Intensity*			
Happy	0.873	.355	.017
Angry	0.127	.724	.003
*Saliency*			
Happy	2.446	.124	.047
Angry	0.966	.330	.019

*N* = 52. *Note.* *p <.05.

**Fig 4:**
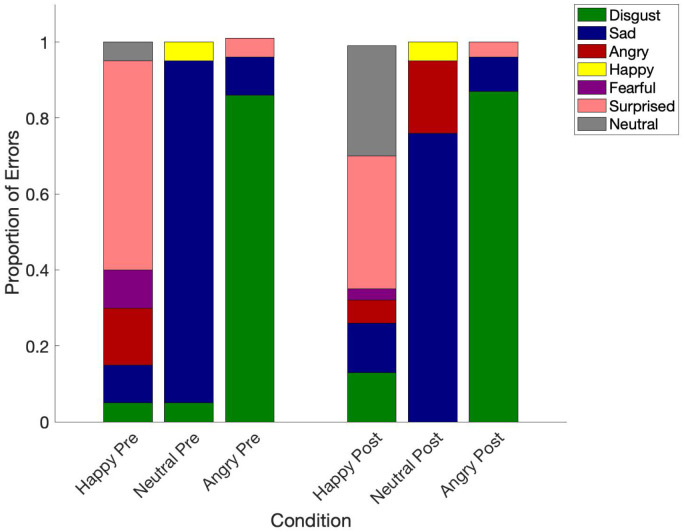
Proportion of errors for each emotional facial expressions category indicating types of errors made by participants.

Additional exploratory analyses using one-way ANOVAs were conducted to assess FER performance across the pre- and post-mood induction conditions. Post hoc tests were adjusted using a Bonferroni correction. The results highlight that participants were significantly worse at recognizing neutral and angry facial expressions compared to happy facial expressions in both pre- and post-conversation conditions (*F*(1.512, 155.685) = 409.974, **p* *< .001, η_p_^2^ = .799). A distribution of the types of errors made for both pre- and post- happy, angry, and neutral expressions can be found in Fig 4. Similarly, participants rated angry expressions significantly less intensely (*F*(1, 103) = 187.279, **p* *< .001, η_p_^2^ = .645) and saliently (*F*(1, 103) = 416.283, **p* *< .001, η_p_^2^ = .802) as compared to happy facial expressions across all conditions (see Table 4).

**Table 4:** Main effect estimates for emotion recognition task collapsed across time points.

Outcome measures	*M(SD)*_*happy*_	*M(SD)*_*angry*_	*M(SD)*_*neutral*_	*F*	*df*	*η*_*p*_^*2*^
Accuracy	0.910(0.085)	0.61(0.167)	0.244(0.244)	4709.974***	1.512, 155.685	.799
Intensity	74.266(15.093)	54.545(13.739)	–	187.279***	1, 103	.645
Saliency	0.840(0.123)	0.588(0.158)	–	416.283***	1, 103	.802

*N* = 52. *Note.* *p <.05; **p <.01; ***p <.001.

### Behavioural ratings

**Fig 5:**
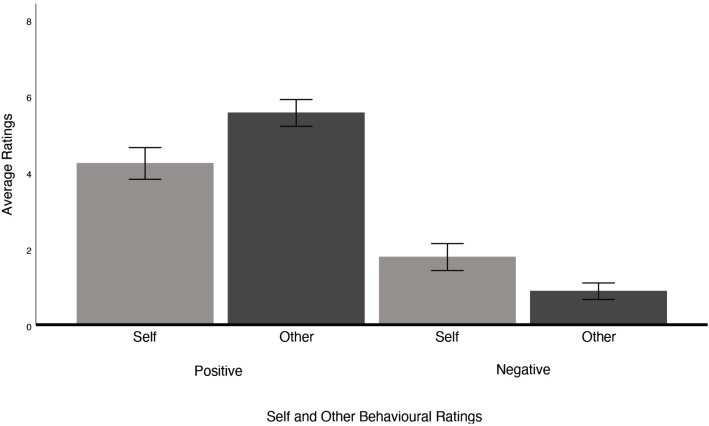
Self and other behavioural ratings. Mean ratings are encompassed by 95% confidence intervals.

Participants were asked to self-assess their perceived performance and the perceived performance of the confederate immediately following the discussion. The results indicate a significant difference across self and other-rated performance for both positive and negative behavioural statements (*F*(1.739, 88.695) = 157.887, **p* *< .001, η_p_^2^ = .756). Post-hoc analyses using a Bonferroni adjustment revealed that participants rated themselves as displaying significantly greater negative characteristics (**p* *< .001) and significantly fewer positive characteristics (**p* *< .001) compared to the confederate (see Fig 5).

### Video ratings

Averaged across both raters, the overall quality of the videos was judged as good to very good. On the SPRS, the raters determined that the confederate maintained very good eye contact (**M* *= 4.71, *SD* = 0.52), good vocal quality (**M* *= 4.47, *SD* = 0.86), spoke at an appropriate length without monopolizing the conversation (**M* *= 4.49, *SD* = 0.86), demonstrated good gesturing and displayed minimal discomfort (**M* *= 4.44, *SD* = 0.67), and consistently maintained good conversational flow with the participants (**M* *= 4.49, *SD* = 0.80).

### Credibility check

After being debriefed at the end of the study, participants were asked how much they believed their responses to the questions were being evaluated by the confederate on a scale of 0–100 (0 = I did not believe it at all, 100 = I completely believed it). Our results indicated that believability was quite high (**M* *= 76.846, *SD *= 25.080).

## Discussion

The goal of this study was to evaluate whether emotional facial expression recognition abilities would be affected by state anxiety in a group of individuals with high trait SA. We aimed to achieve this by asking participants to engage in a pre-post emotion recognition task, which was separated by a mock socially evaluative discussion with a confederate. Given previous research, we hypothesized that our manipulation would be effective in temporarily increasing state anxiety. We anticipated that participants would report greater subjective anticipatory anxiety compared to baseline. Our results suggest that our manipulation was largely ineffective at significantly increasing state anxiety.

There are at least two reasons why we may have been unsuccessful in manipulating state anxiety. First, our confederate was trained to initiate the conversation, and to appear warm and interested. According to emotional contagion theory, peoples’ affective states and emotional reactions can be spread to others [3,43]. Our confederate’s warm and interested style, which was displayed via facial expressions, tone of voice and body posture may have been “contagious,” therefore impacting the emotional state of the participant. In Kelly-Turner and Radomsky (2020), the authors instructed their confederate to initially appear warm and interested, and then switch to appearing cold and disinterested. Following a similar approach may have been beneficial in inducing state anxiety. However, it is equally possible that the confederate’s style had little influence on inducing state anxiety. Studies have shown that a confederate’s specific style (e.g., warm, anxious, flat) when interacting with a participant has no significant influence on modulating state anxiety. Rather, studies suggest that the conversation task alone may be sufficient to induce state anxiety irrespective of the confederate’s behaviour [44].

A second possibility is the duration of the conversation. Previous studies investigating the role of state anxiety on various tasks within a socially anxious sample had participants engage in a 10-minute conversation task [26,38,45]. In a study evaluating how long mood induction procedures last, Gillies and Dozois (2021) note that 7-minute mood induction procedures are an optimal duration, often inducing a negative state lasting between 4–8 minutes [46]. Therefore, having a shorter than recommended mood induction duration may have produced an especially short-lived altered state of anxiety.

Given that our manipulation was ineffective, we were unable to truly test our hypotheses regarding the impact of state anxiety on emotional facial expression recognition abilities. Nevertheless, we did observe that individuals with high trait SA were significantly worse at accurately recognizing happy facial expressions following slight elevations in state anxiety. Here, individuals with SA may be making inferences about others’ emotional expressions that are based on their own anxious state [3], leading to incorrect interpretations about the expressor’s emotional facial expressions. In the post-mood induction condition, happy facial expressions were most often miscategorized as neutral or negative. Yoon and Zinbarg (2007) [47] similarly found that individuals with high trait SA had a tendency to interpret happy facial expressions as disgusted, mocking, or derisive. Similarly, Gutierrez-Garcia and Calvo (2016) found that individuals with SAD mistrust the genuineness of happy facial expressions. Unsurprisingly, we did not observe any additional significant differences in accuracy, intensity, or saliency between our pre- and post-conversation conditions. Similar to previous research, Mullins and Duke (2004) [48] note that in contexts with low situational anxiety, individuals with social anxiety perform just as well in pre-post emotion recognition tasks compared to situations that induce high arousal.

Despite being unable to adequately test our primary hypothesis, we did observe significantly different emotion recognition abilities across conditions. Consistent with other findings, our sample with high trait social anxiety had significantly greater difficulty accurately recognizing neutral facial expressions [15]. Neutral facial expressions were also most often confused with other expressions. A frequency analysis of the incorrectly categorized neutral expressions indicated that neutral expressions were most often confused with sadness. This aligns with cognitive models of SAD, which suggest that individuals with social anxiety demonstrate a negative interpretative bias towards ambiguous facial expressions [49]. Similarly, angry facial expressions were also frequently confused with other negative emotional facial expressions, like disgust. According to Aan Het Rot and colleagues (2022), individuals with high social anxiety likely confuse anger with disgust (and vice versa) because they both elicit similar interpersonal responses. Emotional facial expressions of anger or disgust can both suggest negative evaluation or conflict and therefore result in avoidance [50].

Finally, we did find support for our hypothesis regarding self-evaluations and other-evaluations on the behaviour checklist. Participants rated their own performance during the mock discussion as having *more negative* and *less positive* characteristics compared to the confederate. This is consistent with findings that individuals with social anxiety hold biased perceptions about their own performance in social situations, which likely exacerbates anxiety [40]. However, contrary to Stopa and Clark (1993), participants within our sample rated their own performance as having more positive relative to negative characteristics overall. This result contradicts their hypothesis that individuals with social anxiety tend to underestimate their social abilities. Although our sample had clinically elevated levels of social anxiety on the LSAS-SR, we may have been unable to observe a similar finding due to sampling a non-clinical group of individuals.

## Conclusion

There are a few notable limitations in this study, including duration of the manipulation and no comparable control group. As previously stated, our manipulation was ineffective at significantly increasing state anxiety. Future studies should aim to replicate this study with a greater discussion time, which would allow sufficient time to have the confederate switch to a more disinterested and cold interpersonal style. Following such a protocol may lead to better success in activating fears of negative evaluation, and therefore increasing state anxiety. Insufficient elevations in state anxiety may in part explain why we did not observe significant pre-post differences for many of our outcome measures. However, another contributing factor may be our slightly underpowered analyses. According, to our power analysis, a sample size of **n* *= 54 was required, however, only the data of *n* = 52 were included. Therefore, the low power limits the interpretability of our results. Another noteworthy limitation is the absence of a “control” group. The aim of this study was to evaluate the impact of state anxiety on emotional facial expression recognition abilities within a sample of individuals with high trait social anxiety in a pre-post design. Although we were able to observe clear pre-post differences in accuracy for happy facial expressions, and emotional facial expression recognition differences between happy, angry, and neutral facial expressions overall, it is unclear whether these responses would differ from a group with low trait social anxiety.

With respect to diversity considerations, all participants were recruited through the University of Ottawa’s Integrated System for Participation in Research. Having a university sample may limit generalizability of the results. Future research should attempt to replicate this study with samples collected from more diverse sources, including a clinical sample. As a final note, the Subjective Units of Distress Scale is a single-item inventory and the only measure used in this study to evaluate state anxiety. Incorporating additional measures, like the widely used 40-item State-Trait Anxiety Inventory may have provided additional insight into understanding the construct of state anxiety [51]. Similarly, including multiple measures that have been shown to correlate with measures of state anxiety and trait social anxiety, such as the Positive and Negative Affect Scales (PANAS) and the Tellegen Multidimensional Personality Questionnaire, would have increased convergent validity [52,53]. Future research should replicate this study implementing the suggested changes listed.
